# Bilateral Hypothenar Hammer Syndrome Case Presentation and Literature Review

**DOI:** 10.1155/2022/2078772

**Published:** 2022-06-28

**Authors:** Andrés-Trasahedo Estíbaliz, Labrador-Sánchez Eztizen, Salazar-Asencio Osman-Alberto, Flores-Robles Bryan-Josué, Pinillos-Aransay Valvanera, Laura Torrecilla Lerena, López-Martín Juan-Antonio

**Affiliations:** ^1^Rheumatology Department, San Pedro Hospital, Logroño, La Rioja, Spain; ^2^Neurosurgery Department, Son Espases University Hospital, Islas Baleares, Spain

## Abstract

Hypothenar hammer syndrome is a rare cause of vascular insufficiency. Generally, patients report a history of repetitive trauma to the hypothenar region of the hand. Symptoms often consist of cold intolerance, pain, paleness, and paresthesia due to digital ischemia. The severity of these symptoms will depend on the extent of ulnar artery occlusion and the presence or absence of collaterals between this artery's superficial and deep branches. It is a rare clinical entity, which on multiple occasions requires a surgical approach. We present a 63-year-old man with bilateral Raynaud's phenomenon secondary to hypothenar hammer syndrome successfully treated by vascular repair surgery. In patients with Raynaud's phenomenon, it is important to know that there are reversible causes such as hypothenar hammer syndrome.

## 1. Introduction

First described by Van Rosen in 1934, hypothenar hammer syndrome is an infrequent cause of Raynaud's phenomenon [1]. It usually occurs due to repeated microtraumas on the hypothenar eminence of the hand [2, 3]. The hook of the hamate strikes the superficial palmar branch of the ulnar artery in the Guyon space, leading to the development of (1) an aneurysmal dilation of the artery with subsequent distal embolization and (2) a local segmental occlusion of the ulnar artery [1]. Clinically, it is characterized by pain, intolerance to cold and cyanosis, paresthesias, digital ulcers, or palpable mass [1]. The gold standard for diagnosis is angiography since it provides information on the lesion's location, segment, and extent [1].

## 2. Case Presentation

A 63-year-old man, a medical doctor, and a cycling fan consulted for an episode of paleness and coldness affecting the fourth finger of the right hand, lasting three days. The patient reported that two months ago, he had presented the appearance of a tumor in the hypothenar region of the right hand. During the months of confinement due to the pandemic, he had been cycling as a sports activity. However, he did not report any symptoms suggestive of connective tissue disease.

His personal history included hypertension, dyslipidemia, and subarachnoid hemorrhage at age forty-five. He denied the use of tobacco, alcohol, or other toxic substances and was on regular treatment with aspirin, atorvastatin, ezetimibe, and telmisartan. Blood pressure was normal in both arms on physical examination, and cardiopulmonary auscultation did not show any relevant findings. Pulses in all four limbs were symmetrically palpable.

An analytical study including hemogram, biochemical profile, coagulation study, and autoimmune serology (ANA, ANCA, antiphospholipid antibodies) was performed, with normal values. In addition, an ultrasound study was performed in the vascular laboratory of the infrarenal aorta, common iliac arteries, and popliteal arteries, which was also normal.

Magnetic resonance angiography was performed on both hands, showing an aneurysm in the distal area of the ulnar artery (approximately 7 mm in diameter in the left hand and an aneurysm of 4 mm in diameter in the right hand).

**Figure 1:**
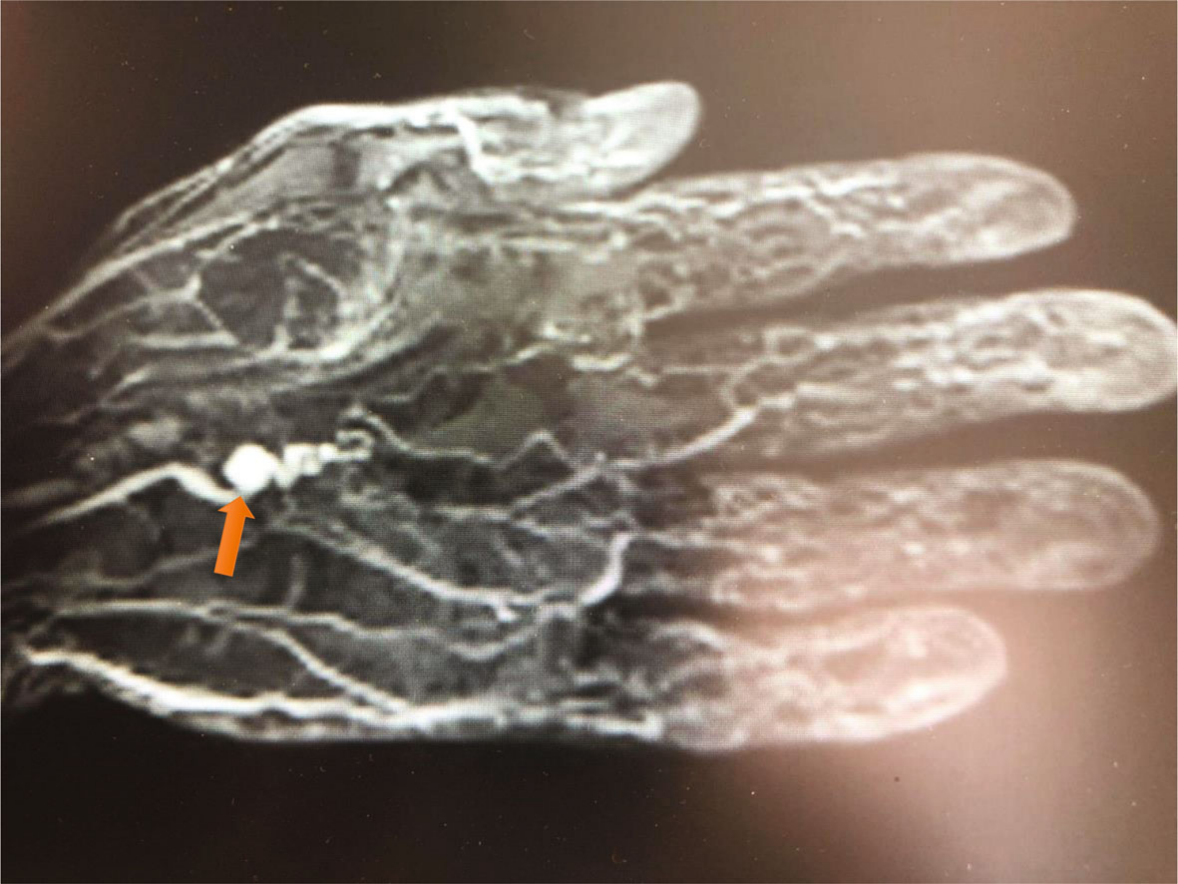
Angioresonance showing an aneurysm approximately 7 mm in diameter in the distal area of the ulnar artery, typical “corkscrew” image (red arrow).

**Figure 2:**
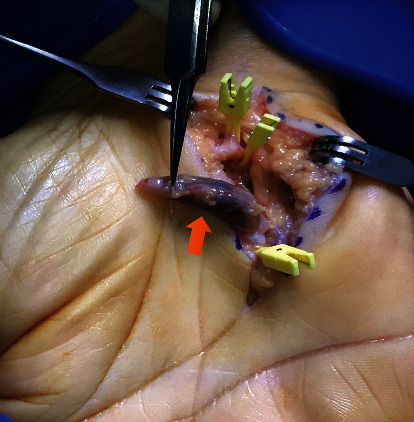
Vascular surgery repair with vein graft. The image shows the aneurysm of the ulnar artery (red arrow).

In both hands and distal to the aneurysm, a tortuous course of the ulnar artery referred to as a “corkscrew image” was observed, this being a typical finding of hypothenar hammer syndrome (Figure 1). The patient was referred to surgery and underwent vascular repair surgery with a vein graft in both hands (Figure 2). Four months after surgery, he is asymptomatic.

## 3. Discussion

Hypothenar hammer syndrome is one of the reversible causes of Raynaud's phenomenon [4, 5]. Its exact incidence is unknown and predominantly affects males [5]. It is common in people who use the volar surface of the hand as a hammer, although this history is not always documented [6]. It has also been linked to certain hobbies such as cycling, sport practiced by the patient. Repeated trauma to the hypothenar eminence of the hand leads to damage to the wall of the ulnar artery, with the consequent aneurysmal formation [2, 3]. Although it is usually unilateral, affecting the dominant hand, cases of bilateral involvement have also been described [2–18] (Table 1); in the case of the patient, the symptomatic hand was the right, although the left was asymptomatic, it presented the typical corkscrew lesion in the angiographic study. The presentation of symptoms is usually intermittent and persists for years. To date, its etiology is unknown, but it has been proposed that it is due to a preexisting abnormality in the ulnar artery, making it particularly sensitive to repetitive microtrauma damage [5].

The most commonly affected fingers are the third and fourth [7], like our case. Clinically, it is characterized by pain, intolerance to cold and cyanosis, paresthesias, digital ulcers, or palpable mass being less frequent (as in our case) [11]. The severity of symptoms depends on the extent of the occluded vascular segment and collateral circulation [8]. Regarding the diagnosis, the Allen maneuver is usually positive in most cases [7]. However, the gold standard for diagnosis is angiography since it provides information on the lesion's location, segment, and extent [1, 9]. Another technique that can be done is Doppler ultrasound that is used to assess blood flow and site of thrombus formation in the ulnar artery and the superficial palmar arch. This test is convenient but requires operator experience to carefully map the arterial tree, and the patient must avoid movement during the entire examination. The benefit of Doppler mapping is that it does not induce vasospasm and may provide a demonstration of arterial hemodynamics [19].

The optimal treatment has not been determined to date since it is a rare disease, making challenging a comparison between different approaches [10–13]. Initially, conservative treatment is recommended, including calcium channel antagonists, beta-blockers, and botulinum toxin, among others [8]. The surgical approach is recommended if there are severe signs or symptoms [5]. Often decisions regarding surgical versus nonsurgical treatments are based on the acuteness of the symptoms and severity of the ischemia. Surgical options in this setting include the following: (1) arterial ligation (assuming an intact radial/palmar arch), (2) resection of thrombosed arterial segment or aneurysm with end-to-end anastomosis, and (3) resection and vascular reconstruction with vein or artery graft [5].

Surgery is not usually indicated for asymptomatic ulnar artery occlusion [5]. However, surgery is indicated in patients who present a “corkscrew” image on angiography with or without symptoms of digital ischemia to prevent the onset/recurrence of symptoms [5] like this case.

In conclusion, a patient with a bilateral hypothenar hammer syndrome is described as an uncommon cause of digital ischemia, thus demonstrating the importance of taking this clinical entity into account as a cause of reversible Raynaud's phenomenon.

## Figures and Tables

**Table 1:** Literature review of bilateral Hypothenar hammer syndrome Cases [2–18].

Author (year)	Cases	Treatment
Williams et al. [4]	1	Vascular surgery
Wernick et al. [9]	1	Vascular surgery
Bhatla et al. [7]	1	Vascular surgery
Neill-Cage et al. [2]	1	Vascular surgery
Lambert et al. [3]	1	Vascular surgery
Ferris et al. [5]	12	Vascular surgery
Dethmers et al. [10]	2	Vascular surgery
Abudakka et al. [11]	1	Amputation
Facca et al. [12]	1	Vascular surgery
Temming et al. [13]	1	Vascular surgery
Larsen et al. [14]	6	Vascular surgery
Scharnbacher et al. [6]	5	No described
Sükrü et al. [8]	3	Vascular surgery
Niet et al. [15]	1	Vascular surgery
Endress et al. [16]	2	Vascular surgery
Kaji et al. [17]	5	Not described
Jin et al. [18]	1	Symptomatic
Flores-Robles et al. (2021)	1	Vascular surgery

## Data Availability

No supporting data is available.
